# A simple, sensitive and rapid isocratic reversed-phase high-performance liquid chromatography method for determination and stability study of curcumin in pharmaceutical samples

**Published:** 2017

**Authors:** Farjad Amanolahi, Ali Mohammadi, Reza Kazemi Oskuee, Hooriyeh Nassirli, Bizhan Malaekeh-Nikouei

**Affiliations:** 1 *Department of Drug and Food Control, School of Pharmacy, Tehran University of Medical Science, Tehran, Iran*; 2 *Department of Medical Biotechnology, School of Medicine, Mashhad University of Medical Sciences, Mashhad, Iran*; 3 *Pharmaceutical Research Center, School of Pharmacy, Mashhad University of Medical Sciences, Mashhad, Iran*; 4 *Nanotechnology Research Center, School of Pharmacy, Mashhad University of Medical Sciences, Mashhad, Iran*

**Keywords:** Curcumin, Hydrolysis, Photostability, RP-HPLC

## Abstract

**Objective:**

This study was designed to develop and validate a new reversed-phase high-performance liquid chromatography (RP-HPLC) method based on Q_2 _(R_1_) International Conference on Harmonization (ICH) guideline for determination of curcumin in pharmaceutical samples.

**Materials and Methods:**

The HPLC instrument method was optimized with isocratic elution with acetonitrile: ammonium acetate (45:55, v/v, pH 3.5), C18 column (150 mm×4.6 mm×5 µm particle size) and a flow rate of 1 ml/min in ambient condition and total retention time of 17 min. The volume of injection was set at 20 µl and detection was recorded at 425 nm. The robustness of the method was examined by changing the mobile phase composition, mobile phase pH, and flow rate.

**Results:**

The method was validated with respect to precision, accuracy and linearity in a concentration range of 2-100 µg/ml. The limit of detection (LOD) and limit of quantification (LOQ) were 0.25 and 0.5 µg/ml, respectively. The percentage of recovery was 98.9 to 100.5 with relative standard deviation (RSD) < 0.638%.

**Conclusion:**

The method was found to be simple, sensitive and rapid for determination of curcumin in pharmaceutical samples and had enough sensitivity to detect degradation product of curcumin produced under photolysis and hydrolysis stress condition.

## Introduction

**Figure 1:**
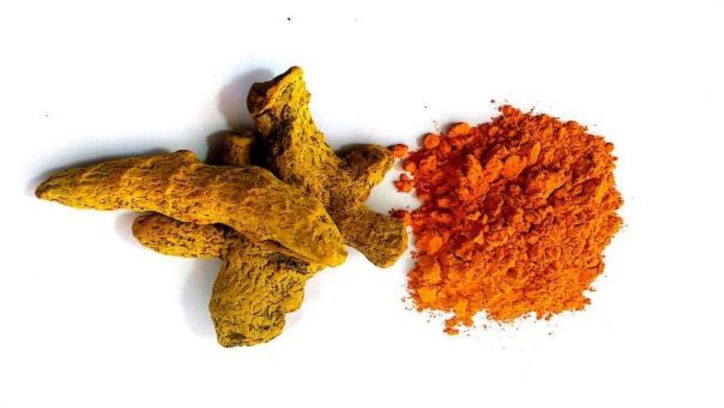
Rhizomes of *Curcuma longa* and curcumin

Today, scientists all over the world, have similar interest in finding a new way to treat cancer using treatments that have the lowest side effects. Curcumin (Figure 1) [1,7-bis (4-hydroy-3-methoxyphenyl)-1,6-heptadiene-3,5-dione], the most active compound of *Curcuma longa* (“Zardchoubeh” in Farsi), from Zingiberaceae family, is one of the widely used medicinal plants in India and other Asian countries. The rhizomes of *C. longa* have been extensively used in India and Southeast Asia and also they are regarded as a spice and coloring agent in cuisine (Aggarwal et al., 2005 ▶).

 Clinical usage of curcumin is justified by its several pharmacological effects such as antioxidant, anti-inflammatory, anti-parasitic, anti-mutagenic, chemoprotective, anti-viral and tumor-preventive properties (Chattopadhyay et al., 2004 ▶ and Aggarwal and Harikumar, 2009 ▶). Today, development of pharmaceutical formulations containing curcumin is limited as curcumin has a low bioavailability. Curcumin has a polyphenolic chemical structure that causes low solubility in water (Ammon and Wahl, 1991 ▶). This compound showed low oral bioavailability and rapid systemic elimination (Anand et al., 2008 ▶ and Zhou et al., 2011). 

 Because of this problem and in order to improve pharmacokinetic profile of curcumin, several new drug delivery systems such as liposome, solid nanoparticles, poly (lactide-co-glycolide) (PLGA) and water-soluble nanoparticles have been investigated (Anand et al., 2010 ▶; Kim et al., 2011 ▶ and Ankola et al., 2009 ▶). Beside the new drug delivery system development, an analytical method is needed to detect curcumin in pharmaceutical products.

 Several high performance liquid chromatography (HPLC) methods with different retention times and solvents were suggested for detecting and quantifying curcumin in *C. longa* extract or pharmaceutical samples. But, it is very important to have a method with high precision and accuracy, with the lowest retention time. The first research for development of a validated HPLC method was reported in 1985 (Tonnesen and Karlsen, 1985 ▶). This method used methanol and water with a retention time of 30 min. In 1988, in an improved method (Roussef, 1988 ▶), curcumin was determined on ODS column using a gradient water-tetrahydrofuran system, within 22 min. Also, another study has reported a HPLC-UV method for determination of curcumin in *C. longa* extract with a gradient system of solvents consisting of methanol, acetic acid and acetonitrile (ACN) with a retention time of 20 min (Jayaprakasha et al., 2002 ▶).

**Figure 2:**
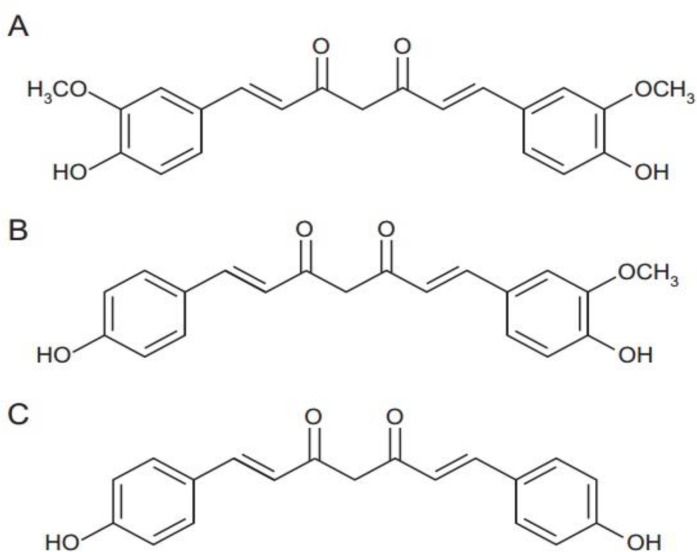
Chemical structures of curcuminoids

 The extract of *C. longa* is a mixture of 3 different compounds, known as curcuminoids (C_3_ complex of curcumin). The major compound is curcumin and the other two compounds are desmethoxycurcumin (DMC) and bisdesmethoxycurcumin (BDMC) (Figure 2) and dry extract of *C. longa *contains 75 %, 20% and 5% of these compounds, respectively, but their content depends on the method of extraction and purification from *C. longa* (Wichitnithad et al., 2009 ▶). 

 Another problem is curcumin’s rapid photodegradation. Tonnesen studied the photodecomposition products and showed that vanillic, vanillin and ferulic acids were the most abundant products of curcumin decomposition in liquid and solid pharmaceutical samples (Tonnesen et al., 1986 ▶).

 Curcumin is stable in acidic pH but two groups of ketone in the structure of curcumin cause rapid degradation in solutions with alkaline pH (Tonnesen and Karlsen, 1985a ▶). In another study, level of curcumin degradation products increased when placed in 0.1 M phosphate buffer (pH 7.2) at 37 °C and 90% of curcumin was decomposed in 30 min. Curcumin has different degradation products such as vanillin, ferulic acid, feruloyl methane but trans-6-(4-hydroxy-3-methoxyphenyl)-2,4-dioxo-5-hexenal is the major product that produces under stress condition (Wang et al., 1997 ▶).

 As above-mentioned, a valid analytical method should be able to detect degradation products with a high accuracy and precision. The aim of this study was to develop a simple and rapid isocratic HPLC-UV method with high sensitivity to determine curcumin and its degradation products in pharmaceutical samples and the final formulations.

A: Curcumin, B: Demethoxycurcumin and C: Bisdemethoxycurcumin

## Materials and Methods

HPLC grade methanol and acetonitrile (ACN) were purchased from Merck (Germany). Water was deionized and triple distilled. HPLC grade curcumin (>99.8%) was provided from Sigma (Germany). C_3_ complex of curcumin was purchased from Sami-Lab (India). All other chemicals and reagents were of analytical grade.

**Buffer solution stability**

According to stability of curcumin in acidic pH, four buffer solutions with pH 2, 2.5, 3 and 3.5 were prepared with below formulation:

*Buffer solution pH 2.0*

Here, 8.95 g of disodium hydrogen phosphate and 3.4 g of potassium dihydrogen phosphate were dissolved in distilled water (D.W.) and diluted to 1000 ml with the same solvent. pH was adjusted with phosphoric acid.

*Buffer solution pH 2.5*

Here, 4.9 g phosphoric acid was added to 500 ml of D.W. pH was adjusted with 0.1 M sodium hydroxide solution.

*Buffer solution pH 3.0*

Here, 21 g citric acid was dissolved in 200 ml of 1 M sodium hydroxide and diluted to 1000 ml with D.W. Then, 40.3 ml of this solution was diluted to 100 ml with 0.1 M hydrochloric acid.

*Buffer solution pH 3.5*

Here, 25 g ammonium acetate was dissolved in 100 ml D.W. pH was adjusted with 0.01 M hydrochloric acid.

Buffer solutions were kept at 25 °C in the dark and pH of them was measured twice/day for 5 days. 

**HPLC instrument and chromatographic condition**

HPLC was performed using a Shimadzu (Kyoto, Japan), consisting of a SCL-10A controller, LC-10ADVP chromatographic pump and Shimadzu UV-SPD-10AVD spectrophotometric detector. The process of separation was done on Varian chromatography C18 column (150 mm×4.6 mm×5 µm particle size). Mobile phase was programmed using an isocratic system with 1 ml/min flow rate at room temperature. Mobile phase of ACN and 0.1 M ammonium acetate buffer (45:55 % v/v) was adjusted to pH 3.5 by 0.01 M HCl and detection wavelength was 425 nm. All solutions and samples for injection were filtered through a 0.45 µm nylon membrane. The volume of injection was set at 20 µl.

**Preparation of stock and standard solutions**

 For this purpose, 50 mg of curcumin was dissolved in 50 ml methanol to achieve a curcumin concentration of 1000 µg/ml. All solutions were prepared in amber glassware and stored at 4 °C in the dark. Calibration and standard solutions were prepared by diluting the stock solution with methanol to obtain concentrations of 2, 5, 10, 20, 50 and 100 µg/ml of curcumin. 

**Validation of RP-HPLC**

Q_2_ (R_1_) ICH guideline was used for HPLC validation process.

*Precision and accuracy*

A method’s repeatability concerns intra-day precision and inter-day precision. In this study, the method accuracy was determined by three concentrations of curcumin (10, 20, and 50 µg/ml). Intra-day precision and accuracy were checked by six replicates for each concentration. Inter-day precision was examined for each concentration on 3 days. The precision was evaluated by percentage relative standard deviation (%RSD) and accuracy was expressed by the percentage of recovery using below formulation:

$\mathrm{Recovery}(\%)=\frac{\mathrm{Detection Concentration}}{\mathrm{Actual Concentration}}\times 100$

*Linearity*

Six different standard solutions of 2 to 100 µg/ml of curcumin were analyzed three times for each concentration. The analytical responses were recorded and calibration curve was prepared by plotting peak area against sample concentration. The linearity was assessed by calculating the slope, y-intercept and correlation coefficient (r^2^) using least square regression.

*Limit of detection (LOD) and limit of quantification (LOQ)*

LOQ was defined as the lowest concentration of analyte that was reproducibly quantified above baseline following triplicate injection with acceptable intra-assay precision and accuracy. LOD was the smallest concentration detectable by UV-VIS detector. These parameters were calculated based on the standard deviation (SD) of y-intercept and the slope (s) as 3.3 and 10 SD/s for LOD and LOQ, respectively.

$\mathrm{LOD}=10\frac{\mathrm{SD}}{s}$ $\mathrm{LOQ}=3.3\frac{\mathrm{SD}}{s}$

*Robustness*

To study the robustness of the method, some changes were made in the flow rate, pH of mobile phase and mobile phase composition. Ratio of both solvents was changed by ±10%. pH of the buffer and flow rate was changed by ±0.3 unit. Three different concentrations were injected in triplicates. The acceptance criteria was RSD< 2%.

**Application of HPLC method**

To study the selectivity of the method, equal solutions of curcumin were prepared from Sigma sample and C_3_ complex from Sami-Lab at 50 µg/ml in methanol. Then, 20 µl of solutions was injected into HPLC to get their chromatograms.

*Photochemical degradation*

In this step, 10 ml methanolic solution of C_3_ complex at 50 µg/ml was directly exposed to the sunlight for 30 min. Thereafter, 20 μl of the solution was injected into the HPLC for determination of curcumin and photolysis degradation products.

*Hydrolysis degradation*

Solution of C_3_ complex was prepared at 50 µg/ml in PBS buffer (pH=7). After 24 h, 20 μl of the solution was injected into the HPLC for determination curcumin and hydrolysis degradation products.

**Statistical analysis**

All experiment was done in triplicate and data were expressed as the mean ± standard deviation (SD). Statistical analysis were performed by using Prism Software Ver.5. Compression between differences of means was analyzed by one-way ANOVA with p value of 0.05 or less were considered significant. 

## Results

**Buffer stability study**

**Figure 3:**
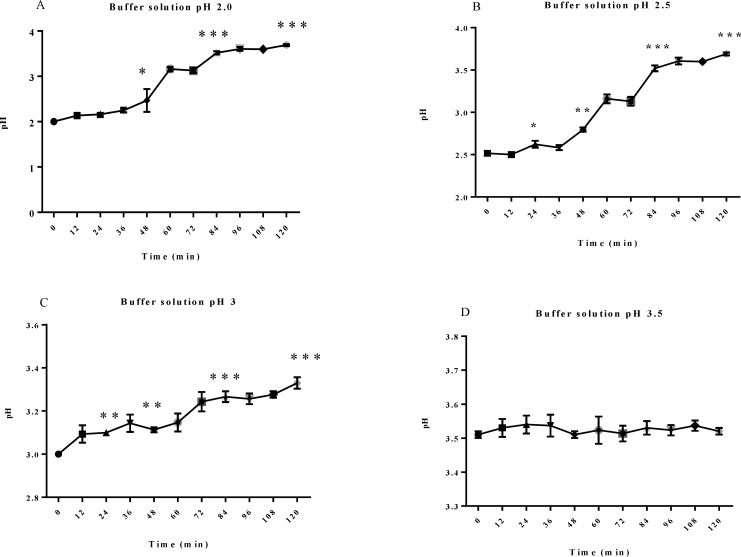
Stability of buffer solution in 5 days, A: pH 2, B: pH 2.5, C: pH 3, D: pH 3.5. (*p< 0.01, **p< 0.001, ***p< 0.000

ANOVA and Sidaks test were used to analyze the pH variation of each buffer during 5 days. The p-value for buffer with pH of 2, 2.5 and 3 was 0.0001 but for pH 3.5 was 0.7432. These results indicate that the buffer with pH 3.5 did not have any significant variation in pH during study (Figure 3). So, 0.1 M ammonium acetate buffer was stable and suitable for HPLC analysis. 

**Table 1.** Precision (% RSD) and accuracy (% recovery) of new RP-HPLC for determination of curcumin

**Repeatability (intra-day), n=6**	**Stock concentration (µg/ml)**	**Amount found** **(µg/ml)**	**% RSD**	**% Recovery**
	10	10.1	0.490	101
**Curcumin**	20	20.1	0.495	101
	50	49.6	0.306	99.2
**Inter-day precision 3 days (n=6)**				
**First Day**				
	10	10.07	0.638	100.7
	20	20.02	0.264	100.1
	50	49.89	0.22	99.78
**Second Day**				
	10	9.89	0.567	98.9
	20	19.87	0.626	99.35
	50	49.87	0.543	99.74
**Third Day**				
	10	9.999	0.11	99.99
	20	20.1	0.246	100.5
	50	50	0.2	100

**Precision and Accuracy**

Term of precision was used to describe an analytical method that has the lowest variation among a series of measurements obtained from several sampling of the same equal sample and the term accuracy is a property of an analytical method that describes the closeness of the results to normal value calculated with percentage of recovery. Table 1 shows the result of intra-day and inter-day precision and recovery of the method. The ranges of %RSD parameters for intra-day precision and inter-day precision were 0.309 to 0.49 and 0.11 to 0.638, respectively. According to the ICH validation guideline, the low value for %RSD (<%2) reflects the high precision of the method. The percentage of recovery for intra-day and inter-day accuracy was 99.2 to 101 and 98.9 to 100.7, respectively. According to the ICH guideline, all results were between 98 and 102 percent suggesting high accuracy of the method.

**Linearity**

**Figure 4:**
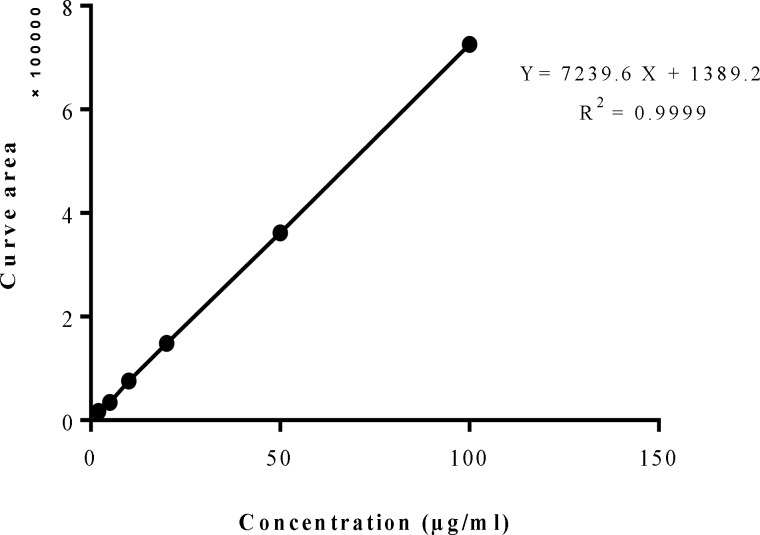
Calibration curve of curcumin at concentrations ranging from 2 to 100 µg/ml

Slope, y-intercept and correlation coefficient (r^2^) of regression analysis were summarized in Table 2. Calibration curve was linear in range of 2 to 100 µg/ml. The regression equation was y= 7239.6x + 1389.2 (r^2^=0.9999) and correlation coefficient was greater than 0.999 that demonstrated a high degree of correlation and good linearity of the method (Figure 4).

**Table 2:** Linear regression data for calibration curve of concentrations ranging from 2 to 100 µg/ml (n= 3

	**Concentration range (µg/ml)**	**Slope**	**y-Intercept**	**r ** ^2^	**p value**
**Curcumin**	2-100	7239.6	1389.2	0.9999	<0.0001

**LOD and LOQ**

The LOD and LOQ were 0.25 µg/ml and 0.5 µg/ml, respectively. This evidence indicates that our method has provided adequate sensitivity.

**Robustness**

Peak area was recorded and %RSD for variation in mobile phase condition, pH of eluent and flow rate for the three concentrations was calculated in triplicates. According to Table 3, %RSD <2 showed that our method was robust with small changes.

**Table 3:** Robustness testing of our method (n=3).

**Parameter**	**Modificaion**	**% RSD**	**% Recovery**
	2.7	0.67	98.9
**pH**	3	0.99	99.6
	3.3	1.1	100.3
			
	50	1.4	100.1
**Buffer**	55	0.92	100.1
	60	1.1	99.3
			
	0.7	0.87	99.1
**Flow Rate**	1	0.89	98.3
	1.3	1.6	100.5

**Analysis C**_3_ **complex**

**Figure 5:**
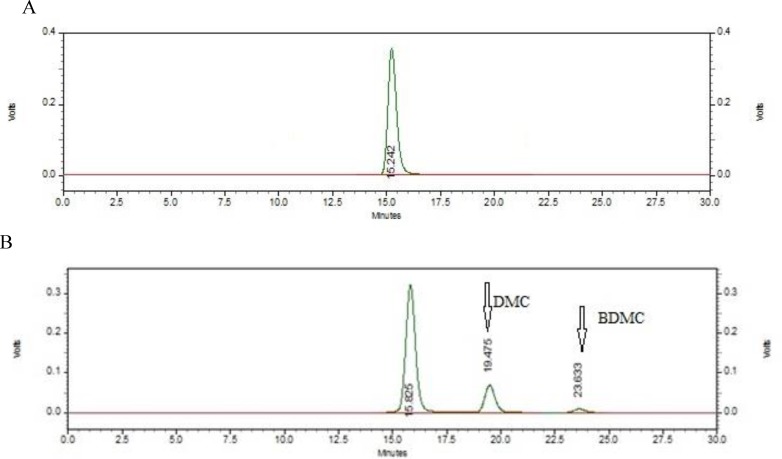
A) Chromatogram of curcumin solution at the concentration of 50 µg/ml (curcumin obtained from Sigma company with >99.8% purity) and (B) C_3_ complex (curcumin obtained from Sami-Lab company

RP-HPLC developed in this study was used to analysis C_3_ complex of curcumin. Figures 5A and 5B show chromatograms of curcumin samples from Sami-Lab and Sigma Company. Good separation of curcumin from other analogues, BDM and DBDM, has indicated high precision and accuracy of the validated method. Table 4 shows the percentage of curcumin and other analogues detected by the new method as compared to certificate of Sami-Lab sample. Our data also demonstrates the high sensitivity of the new RP-HPLC method in detection of curcumin in pharmaceutical samples and finished product.

**Figure 6:**
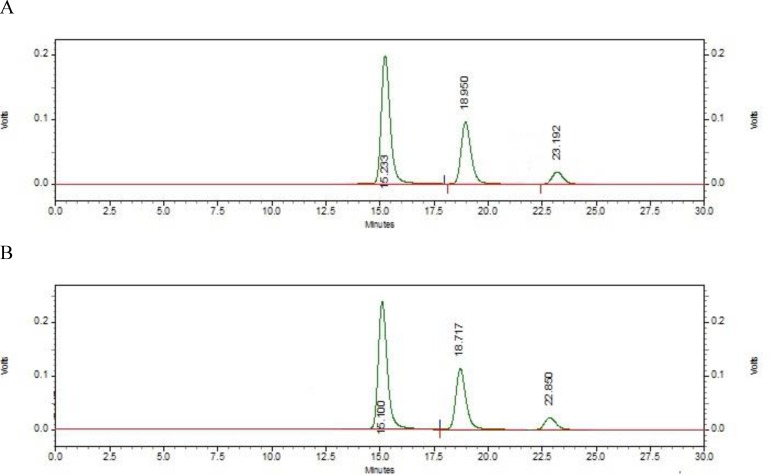
Chromatogram of curcumin solution at the concentration of 50 µg/ml after photolysis and hydrolysis

**Photolysis and hydrolysis stress study**

Figures 6A and 6B show chromatogram of C_3_ complex decomposed after exposure to sunlight (photolysis) and hydrolysis by phosphate-buffered saline (PBS). Data from these chromatograms shows that 38 and 26% of curcumin was degraded under these conditions, respectively and the amount of curcumin was remarkably decreased.

**Table 4:** Amount of curcumin and analogues detected by the validated method

	Curcumin (%)	DMC (%)	BDMC (%)
Sami-Lab certificate	78.19	18.55	3.26
Our method	78.2	18.336	3.206

## Discussion

Curcumin is the major active compound of *C. longa* rhizome, which has been used as a dietary and coloring agent, as well as a medicinal herb. Several studies have been done on pharmacological effects of curcumin such as anti-oxidant, anti-inflammation, and anti-cancer properties (Aggarwal et al., 2005 ▶ and Aggarwal and Harikumar, 2009 ▶).

 As curcumin possesses important pharmacological effects of curcumin and in order to evaluate pharmaceutical formulations containing curcumin such as tablets, capsules or new drug delivery systems such as nanoparticles, it is very important to have a method to quantify and analyze pharmaceutical samples and detect curcumin stability in final formulations.

 In the present study, a new precise, rapid and sensitive reversed-phase HPLC method for determination of curcumin was developed. Results of several studies confirmed that curcumin had low stability when exposed to light and aqueous medium. Therefore, a valid method must be able to separate the major compounds from any degradation products or detect any decrease in the content of the analyte that decompose under stress or during storage. Our method was validated based on ICH guidelines. Q_1_B is referred to the photostability testing and the guideline Q_2_ (R_1_) is about method validation process.

 Several studies have been done to determine curcumin and other curcuminoids in validation or stability studies. In these reports, the retention time of curcumin was more than that of our method (Wichitnithad et al., 2009 ▶ and Koranya et al., 2013 ▶).

 The goal of stability study is to determine the content of analyte after exposure to stress such as acidic or basic pH, oxidation with H_2_O_2 _and photolysis by UV or sunlight. According to the ICH guideline, the amount of sample that decomposes under stress condition would not be more than 20% because of production of some byproducts that are not appeared under normal condition during shelf life. For example, in several stability studies on curcumin, more than 20% of the sample was decomposed and the new byproduct was seen in chromatogram (Koranya et al., 2013 ▶, Dandekar and Patravale, 2009 ▶).

 According to the ICH Q_1_A-R_2 _guideline, sensitive drug substances do not examine in the accelerated storage condition but these compounds should be evaluated in the intermediate condition.

 The novelty of our study was the usage of ammonium-acetate as buffer which caused the appearance of curcumin peak in the first place in chromatogram. Figure 3A shows chromatogram of curcumin from Sigma sample with total retention time of 17 min, but when C_3_ complex of curcumin was used, the retention time increased to 25 min because of the two other analogues, DMC and BDMC. Hence, our new validated methods had enough sensitivity for separation of curcumin from another analogue within an acceptable time period. This selectivity is directly due to the use of ACN in the elution system. Curcumin does not have molecular charge; so for good separation, ACN was used because it is more polar than methanol, and this system was suitable for separation of compounds with low molecular charge and lipophilicity.

 According to the ICH Q_1_A-R_2 _guideline, we exposed curcumin to hydrolysis in neutral pH and photolysis by sunlight. Figures 4A and 4B show a significant decrease in the amount of curcumin, about 38% during photolysis and 26% in hydrolysis testing. This data demonstrated that our method had a high level of selectivity for detection and quantification of curcumin from other degradation products. Also, a significant increase in the area under curve of DMC and BDMC chromatograms, was observed that may be because of curcumin degradation and production of another analogue.

In the present study, an isocratic, simple and precise RP-HPLC method was developed for detecting curcumin. Experimental conditions including mobile phase condition, pH of mobile phase and flow rate were validated in compliance with ICH guideline, Q_2 _(R_1_) 2005. This method was suitable for determination of curcumin in pharmaceutical samples with suitable precision, accuracy and linearity. Therefore, it is suggested that this method has a good potential for routine analysis of curcumin in any samples such as pharmaceutical bulk or turmeric extract.
